# Supplementary material for: Effects of Differences of Breakfast Styles, Such as Japanese and Western Breakfasts, on Eating Habits
Source: Nutrients. 2022 Dec 2;14(23):5143. doi: 10.3390/nu14235143 (PMC9740526; doi:10.3390/nu14235143)
Supplement: Supplementary file 1 [file nutrients-14-05143-s001.zip › Supplemental Figure1-revised.pptx]

## Slide 1
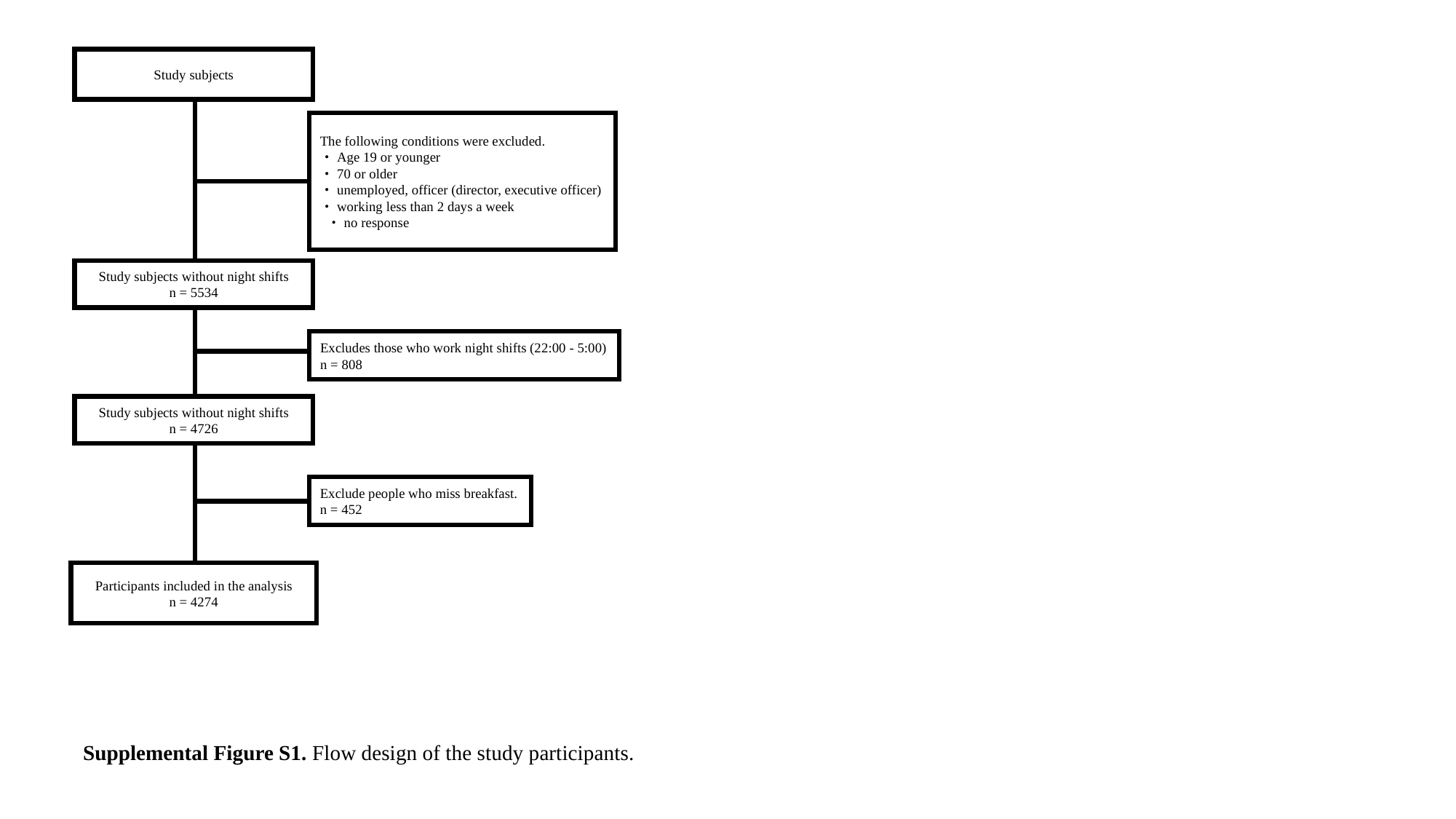

Study subjects
The following conditions were excluded.
・Age 19 or younger
・70 or older
・unemployed, officer (director, executive officer)
・working less than 2 days a week
 ・no response
Study subjects without night shifts
n = 5534
Excludes those who work night shifts (22:00 - 5:00)
n = 808
Study subjects without night shifts
n = 4726
Exclude people who miss breakfast.
n = 452
Participants included in the analysis
n = 4274
Supplemental Figure S1. Flow design of the study participants.
