# Supplementary material for: Effects of Differences of Breakfast Styles, Such as Japanese and Western Breakfasts, on Eating Habits
Source: Nutrients. 2022 Dec 2;14(23):5143. doi: 10.3390/nu14235143 (PMC9740526; doi:10.3390/nu14235143)
Supplement: Supplementary file 1 [file nutrients-14-05143-s001.zip › Supplemental Table1_1026-revised.pptx]

## Slide 1
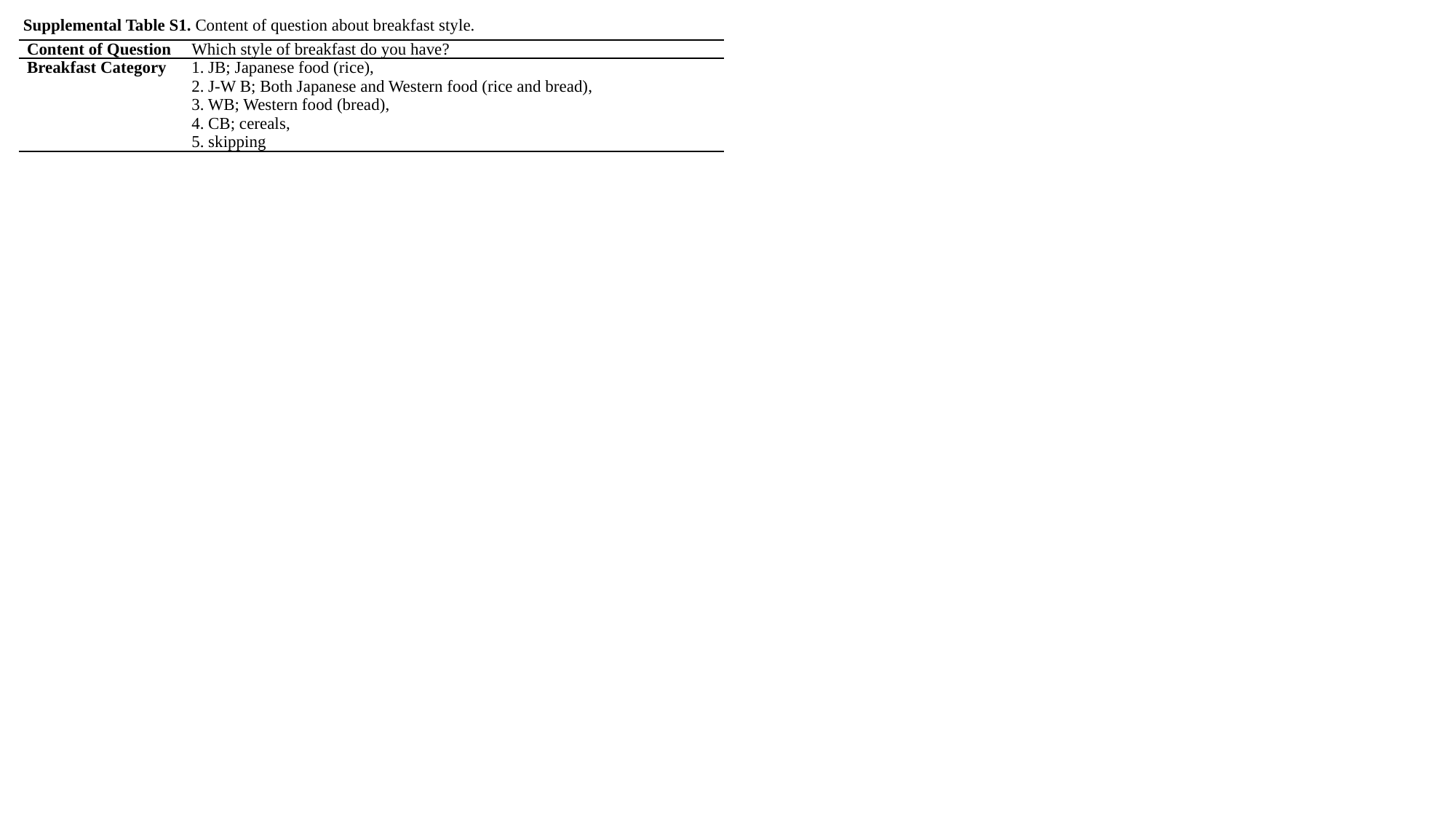

Supplemental Table S1. Content of question about breakfast style.
| Content of Question | Which style of breakfast do you have? |
| --- | --- |
| Breakfast Category | 1. JB; Japanese food (rice), 2. J-W B; Both Japanese and Western food (rice and bread), 3. WB; Western food (bread), 4. CB; cereals, 5. skipping |
