# Supplementary material for: Effects of Differences of Breakfast Styles, Such as Japanese and Western Breakfasts, on Eating Habits
Source: Nutrients. 2022 Dec 2;14(23):5143. doi: 10.3390/nu14235143 (PMC9740526; doi:10.3390/nu14235143)
Supplement: Supplementary file 1 [file nutrients-14-05143-s001.zip › Supplemental Table2_1026-revised.pptx]

## Slide 1
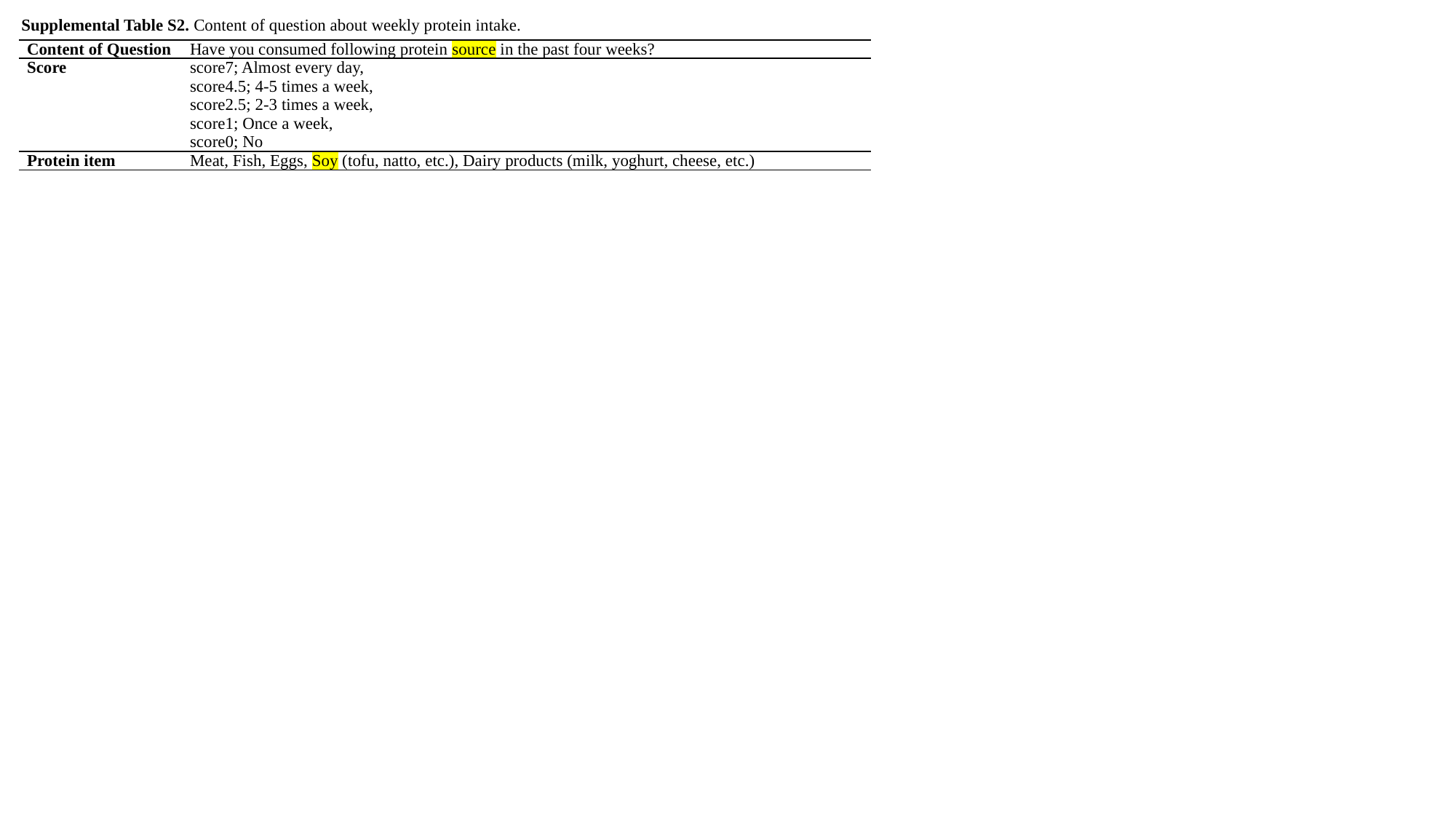

Supplemental Table S2. Content of question about weekly protein intake.
| Content of Question | Have you consumed following protein source in the past four weeks? |
| --- | --- |
| Score | score7; Almost every day, score4.5; 4-5 times a week, score2.5; 2-3 times a week, score1; Once a week, score0; No |
| Protein item | Meat, Fish, Eggs, Soy (tofu, natto, etc.), Dairy products (milk, yoghurt, cheese, etc.) |
