# Supplementary material for: Effects of Differences of Breakfast Styles, Such as Japanese and Western Breakfasts, on Eating Habits
Source: Nutrients. 2022 Dec 2;14(23):5143. doi: 10.3390/nu14235143 (PMC9740526; doi:10.3390/nu14235143)
Supplement: Supplementary file 1 [file nutrients-14-05143-s001.zip › Supplemental Table4_1026-revised.pptx]

## Slide 1
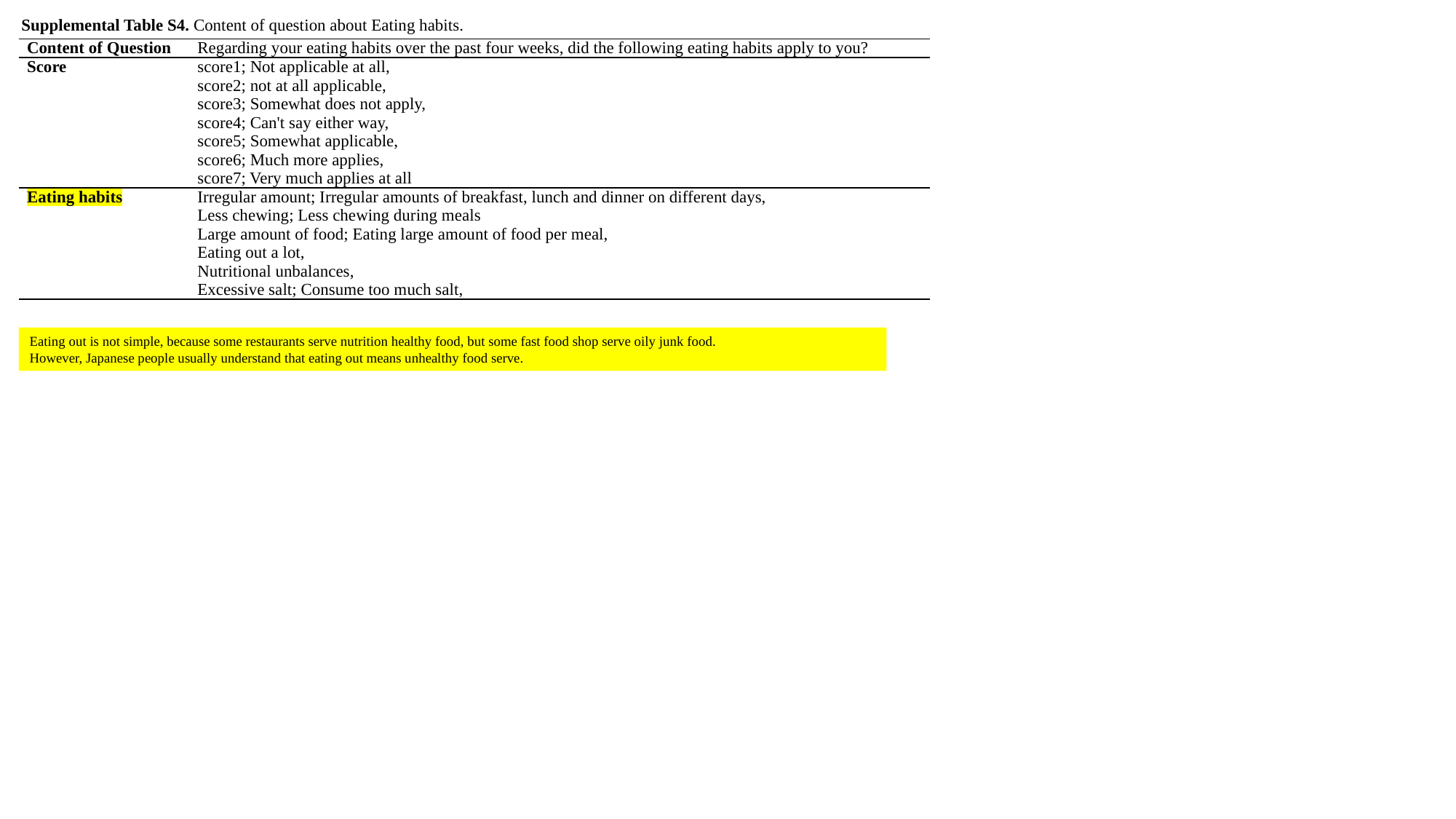

Supplemental Table S4. Content of question about Eating habits.
| Content of Question | Regarding your eating habits over the past four weeks, did the following eating habits apply to you? |
| --- | --- |
| Score | score1; Not applicable at all, score2; not at all applicable, score3; Somewhat does not apply, score4; Can't say either way, score5; Somewhat applicable, score6; Much more applies, score7; Very much applies at all |
| Eating habits | Irregular amount; Irregular amounts of breakfast, lunch and dinner on different days, Less chewing; Less chewing during meals Large amount of food; Eating large amount of food per meal, Eating out a lot, Nutritional unbalances, Excessive salt; Consume too much salt, |
Eating out is not simple, because some restaurants serve nutrition healthy food, but some fast food shop serve oily junk food.
However, Japanese people usually understand that eating out means unhealthy food serve.
