# Supplementary material for: Effects of Differences of Breakfast Styles, Such as Japanese and Western Breakfasts, on Eating Habits
Source: Nutrients. 2022 Dec 2;14(23):5143. doi: 10.3390/nu14235143 (PMC9740526; doi:10.3390/nu14235143)
Supplement: Supplementary file 1 [file nutrients-14-05143-s001.zip › Supplemental Table5_1026-revised.pptx]

## Slide 1
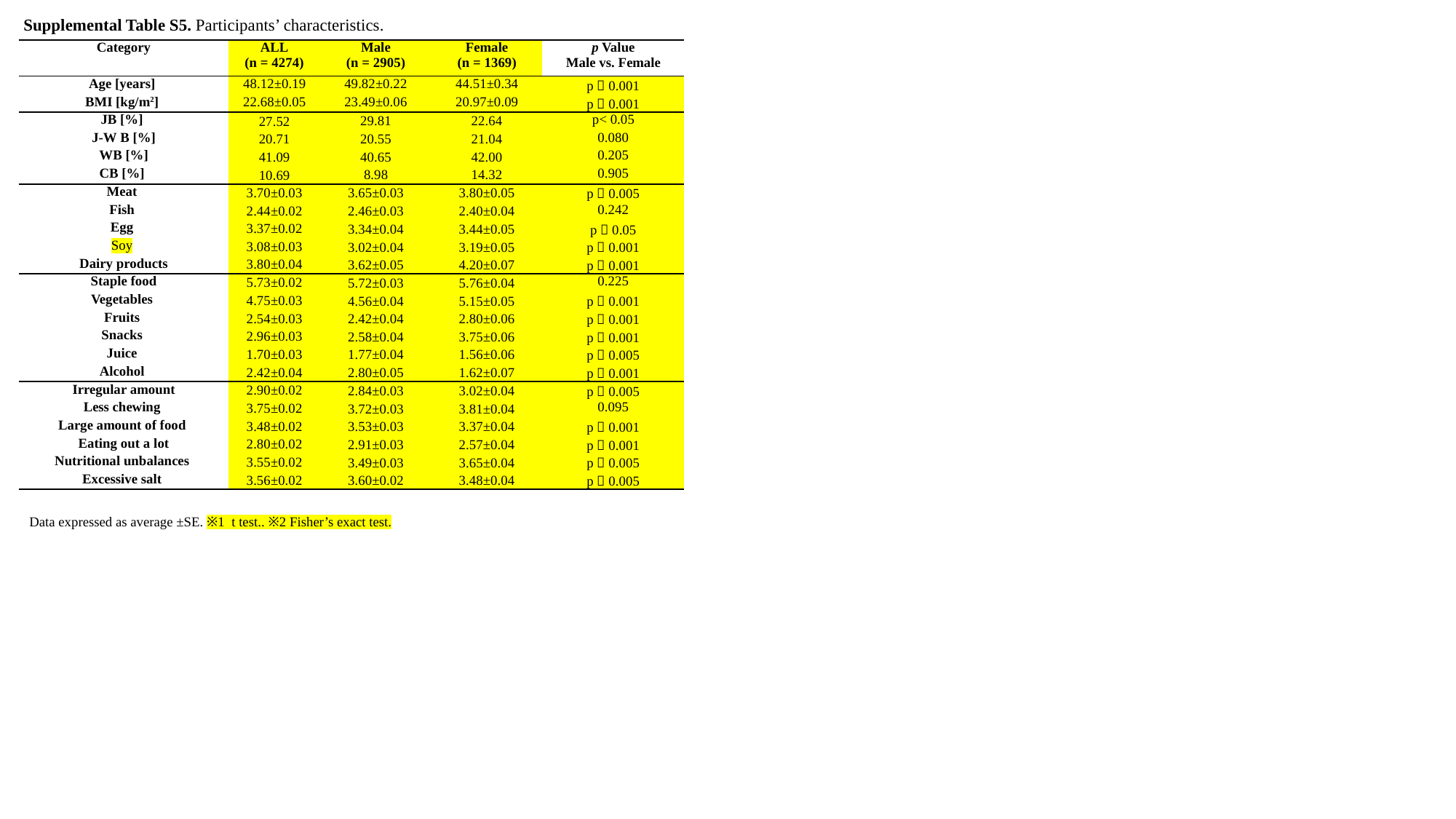

Supplemental Table S5. Participants’ characteristics.
| Category | ALL (n = 4274) | Male (n = 2905) | Female (n = 1369) | p Value Male vs. Female |
| --- | --- | --- | --- | --- |
| Age [years] | 48.12±0.19 | 49.82±0.22 | 44.51±0.34 | p＜0.001 |
| BMI [kg/m2] | 22.68±0.05 | 23.49±0.06 | 20.97±0.09 | p＜0.001 |
| JB [%] | 27.52 | 29.81 | 22.64 | p< 0.05 |
| J-W B [%] | 20.71 | 20.55 | 21.04 | 0.080 |
| WB [%] | 41.09 | 40.65 | 42.00 | 0.205 |
| CB [%] | 10.69 | 8.98 | 14.32 | 0.905 |
| Meat | 3.70±0.03 | 3.65±0.03 | 3.80±0.05 | p＜0.005 |
| Fish | 2.44±0.02 | 2.46±0.03 | 2.40±0.04 | 0.242 |
| Egg | 3.37±0.02 | 3.34±0.04 | 3.44±0.05 | p＜0.05 |
| Soy | 3.08±0.03 | 3.02±0.04 | 3.19±0.05 | p＜0.001 |
| Dairy products | 3.80±0.04 | 3.62±0.05 | 4.20±0.07 | p＜0.001 |
| Staple food | 5.73±0.02 | 5.72±0.03 | 5.76±0.04 | 0.225 |
| Vegetables | 4.75±0.03 | 4.56±0.04 | 5.15±0.05 | p＜0.001 |
| Fruits | 2.54±0.03 | 2.42±0.04 | 2.80±0.06 | p＜0.001 |
| Snacks | 2.96±0.03 | 2.58±0.04 | 3.75±0.06 | p＜0.001 |
| Juice | 1.70±0.03 | 1.77±0.04 | 1.56±0.06 | p＜0.005 |
| Alcohol | 2.42±0.04 | 2.80±0.05 | 1.62±0.07 | p＜0.001 |
| Irregular amount | 2.90±0.02 | 2.84±0.03 | 3.02±0.04 | p＜0.005 |
| Less chewing | 3.75±0.02 | 3.72±0.03 | 3.81±0.04 | 0.095 |
| Large amount of food | 3.48±0.02 | 3.53±0.03 | 3.37±0.04 | p＜0.001 |
| Eating out a lot | 2.80±0.02 | 2.91±0.03 | 2.57±0.04 | p＜0.001 |
| Nutritional unbalances | 3.55±0.02 | 3.49±0.03 | 3.65±0.04 | p＜0.005 |
| Excessive salt | 3.56±0.02 | 3.60±0.02 | 3.48±0.04 | p＜0.005 |
Data expressed as average ±SE. ※1 t test.. ※2 Fisher’s exact test.
